# Genetics and functional significance of the understudied methamphetamine sensitive circadian oscillator (MASCO)

**DOI:** 10.12688/f1000research.125432.2

**Published:** 2022-10-24

**Authors:** S K Tahajjul Taufique, David E Ehichioya, Julie S Pendergast, Shin Yamazaki

**Affiliations:** 1 Department of Neuroscience, UT Southwestern Medical Center, Dallas, Texas, 75390-9111, USA; 2 Department of Biology, University of Kentucky, Lexington, Kentucky, 40506-0225, USA; 3 Peter O’Donnell Jr. Brain Institute, UT Southwestern Medical Center, Dallas, Texas, 75390-8823, USA

**Keywords:** circadian, ultradian, infradian, dopamine, sleep, ADHD, N24SWD, psychostimulant

## Abstract

The last 50 years have witnessed extraordinary discoveries in the field of circadian rhythms. However, there are still several mysteries that remain. One of these chronobiological mysteries is the circadian rhythm that is revealed by administration of stimulant drugs to rodents. Herein we describe the discovery of this circadian rhythm and its underlying oscillator, which is frequently called the methamphetamine-sensitive circadian oscillator, or MASCO. This oscillator is distinct from canonical circadian oscillators because it controls robust activity rhythms independently of the suprachiasmatic nucleus and circadian genes are not essential for its timekeeping. We discuss these fundamental properties of MASCO and integrate studies of strain, sex, and circadian gene mutations on MASCO. The anatomical loci of MASCO are not known, so it has not been possible thus far to discover its novel molecular timekeeping mechanism or its functional significance. However, studies in mutant mice suggest that genetic approaches can be used to identify the neural network involved in the rhythm generation of MASCO. We also discuss parallels between human and rodent studies that support our working hypothesis that a function of MASCO may be to regulate sleep-wake cycles.

## Discovery of the extra-SCN pacemaker that is sensitive to methamphetamine

The mammalian circadian system is most often illustrated as a network of circadian clocks that is organized hierarchically. The primary central circadian pacemaker is in the suprachiasmatic nucleus (SCN) and it receives information from the retina and entrains to the light and dark cycle. Then the SCN coordinates the phases of oscillators in peripheral tissues. The molecular timekeeping mechanism in these circadian clocks is a transcriptional-translational feedback loop of canonical circadian genes (e.g., *Bmal1, Clock, Periods, Cryptochromes*). This conceptualization of the mammalian circadian system has been carefully described many times. ^1^^–^^4^ However, this is not a complete picture of the mammalian circadian system. There are also circadian pacemakers that control circadian rhythms of locomotor activity in rodents, but do not require the SCN or the canonical circadian molecular timekeeping mechanism. These extra-SCN circadian pacemakers are revealed by methamphetamine/amphetamine, restricted food availability (the food-entrainable oscillator or FEO), or other rewarding stimuli such as wheel-running activity and palatable meals. ^5^ It is possible that these extra-SCN pacemakers use the same timekeeping mechanism and even that they are the same oscillator. ^6^ We discussed the similarities of the extra-SCN oscillators in another review article, ^5^ therefore, this review will focus on the mystifying circadian activity rhythm revealed by methamphetamine/amphetamine administration to rodents.

**Figure 1.**
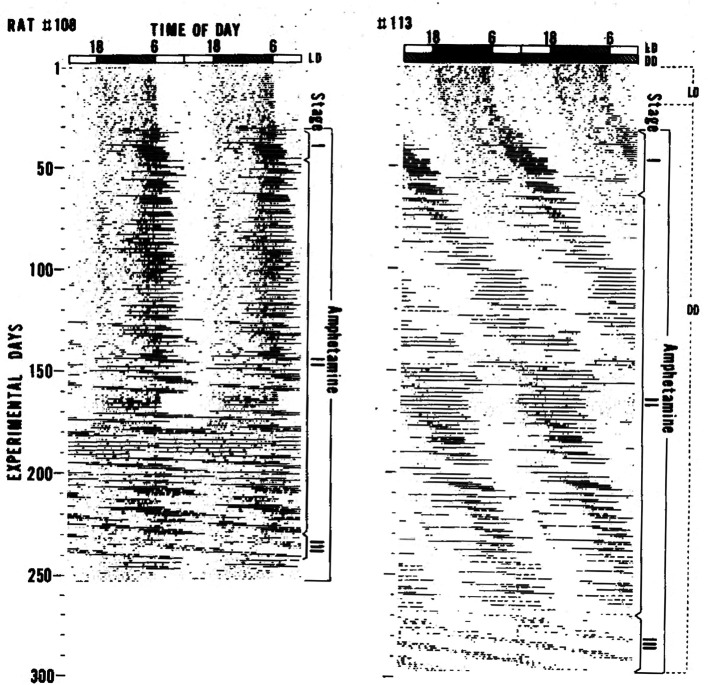
Locomotor activity rhythms in rats are altered by d-amphetamine administered in drinking water. Figure reproduced from Ikeda and Chiba 1982 ^7^ with permission of publisher.

The effect of amphetamine treatment on circadian activity rhythms was first described in 1982 by Ikeda and Chiba. ^7^ They studied the effects of several psychotropics on circadian locomotor activity rhythms in Fischer rats under light and dark conditions (LD). Cocaine, morphine, and FS-32 (a thymoleptic antidepressant discovered by Ikeda) did not robustly affect activity rhythms. In contrast, administering low-dose d-amphetamine (0.01%) in the drinking water had striking and reproducible effects on the locomotor activity rhythm. Upon amphetamine administration, a highly elevated activity bout appeared at the end of activity period (Stage I in Figure 1). This activity bout extended to the beginning of the light phase and then free-ran with a very long period (Stage II in Figure 1). Chronic amphetamine administration caused this newly emerged free-running rhythm to become dominant and the light-entrained activity rhythm controlled by the SCN was no longer visible (Stage III in Figure 1). The periods of the emergent activity rhythms were sometimes in the circabidian (period ~ two days) range of 48–55 h. Ikeda and Chiba observed the same phenomenon when rats were given amphetamine in their drinking water in constant darkness (DD). From those observations, Ikeda and Chiba proposed that there are at least two circadian oscillators underlying the circadian behavior rhythm in rats; one oscillator is more sensitive to amphetamine than the other.

The next seminal discovery was that the psychostimulant-induced activity rhythm was driven by a circadian oscillator located outside of the SCN. Ken-Ichi Honma’s group first showed that low-dose methamphetamine (0.01% or 0.005% in drinking water) had similar effects on activity rhythms in Wistar rats as d-amphetamine treatment in Fischer rats. ^8^ Importantly, they also found that methamphetamine treatment revealed a long-period free-running activity rhythm in SCN-lesioned rats ^9^ indicating that this rhythm is generated by an oscillator outside of the SCN. During the next decade, Honma’s lab established the foundational principles of the methamphetamine-revealed circadian oscillator. In summary, they found that this rhythm dissociated from the SCN-controlled rhythm and free-ran in LD and DD. ^8^ The methamphetamine-revealed rhythm could also entrain to restricted feeding. ^10^ They excluded the possibility that the rhythm was driven by the drinking rhythm by administering methamphetamine via subcutaneous, continuous delivery osmotic pumps. ^9^ Honma and others reported that the methamphetamine-induced rhythm persisted for several cycles after withdrawing methamphetamine/amphetamine administration. ^9^^,^^11^^–^^15^

Three labs named the MA-induced oscillator in the early 2000’s. Ralph’s group named this oscillator the chemically-inducible oscillator (CIO). ^12^ Honma’s group named it the methamphetamine-induced oscillator (MAO). ^16^ Menaker’s group named it the methamphetamine-sensitive circadian oscillator (MASCO). ^11^ These names reflected the distinct conceptualizations of the oscillator by each lab. The names CIO and MAO imply the oscillator is not rhythmic or functional without amphetamine/methamphetamine. MASCO implies the oscillator is functional without, but enhanced by, amphetamine/methamphetamine. Herein we will call the extra-SCN circadian oscillator/pacemaker revealed by amphetamine/methamphetamine MASCO because this name is widely used in recent publications and because we favor the hypothetical functionality implied by this name. There is no established name for the rhythm driven by MASCO. Therefore, we will call it the MASCO-driven rhythm. Storch’s group hypothesized that the MASCO-driven rhythm is the output of a so-called dopamine ultradian oscillator (DUO) whose period is elongated by methamphetamine. ^17^

## Current model of SCN-MASCO coupling

**Figure 2.**
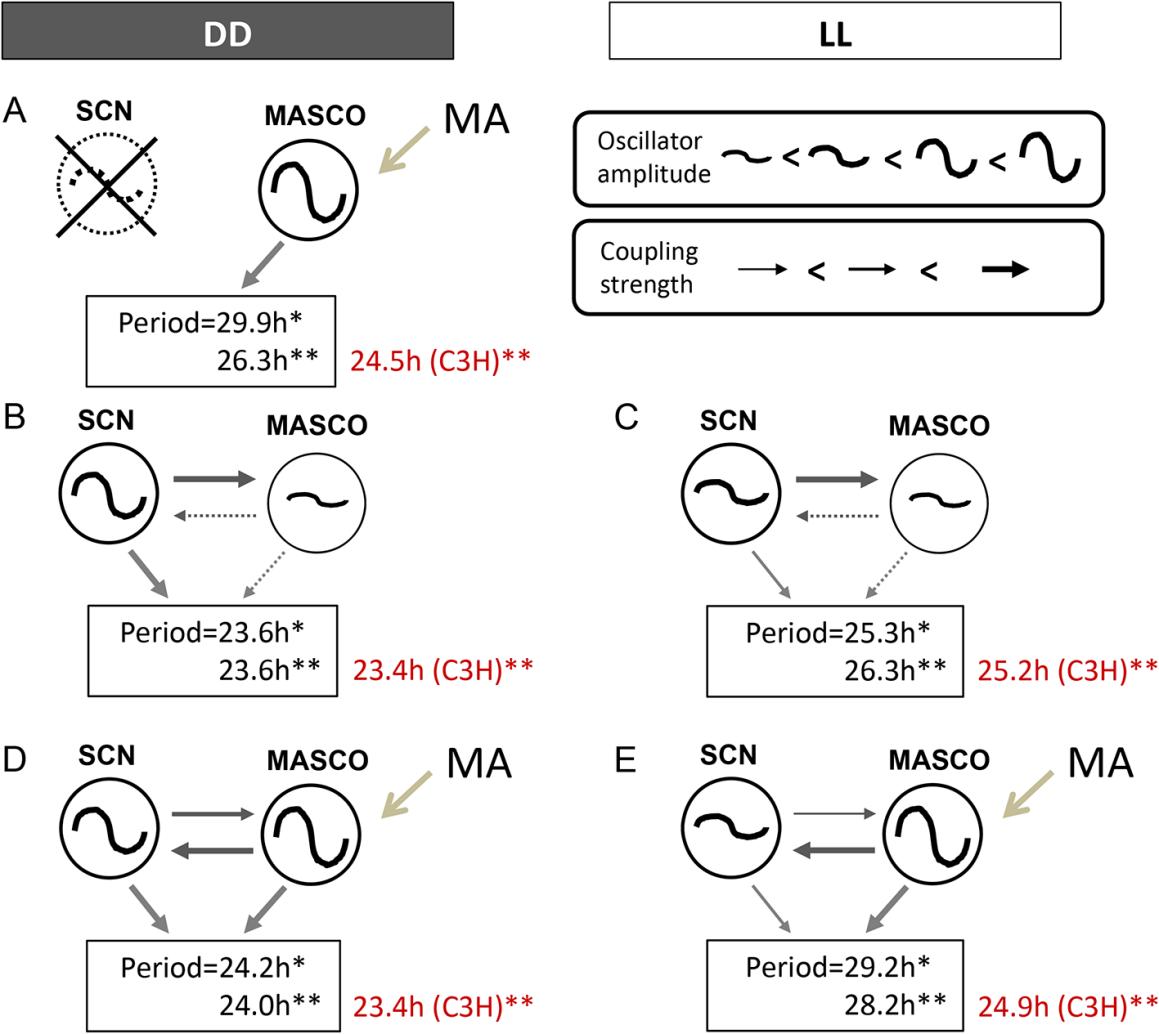
Current model of SCN-MASCO coupling in C57BL/6J mice. The effects of short-term methamphetamine administration on periods of activity rhythms in C57BL/6J (black font) and C3H (red font) mice are shown. Black arrows indicate coupling between oscillators. Gray arrows indicate strength of output. *Data taken from Pendergast *et al*. 2013. **Data taken from Tataroglu *et al*. 2006. See Table 1 for other studies. Figure modified from Yamazaki 2019 ^56^ with permission.

The current working model of MASCO-SCN coupling was developed from studying the periods of activity rhythm(s) in DD and constant light (LL) ( Figure 2A-E, Table 1). The SCN is a robust oscillator in DD, but a much weaker oscillator in LL because the phases of individual cellular oscillators become desynchronized in LL. ^18^ These known differences in SCN oscillator amplitude were used to probe the relationship between the SCN and MASCO. ^11^^,^^19^ The model presented here is based mainly on data collected from C57BL/6J mice, except where indicated ( Figure 2A-E). For ease of discussion, we will refer to methamphetamine treatment, although some studies have been performed with amphetamine and methylphenidate with similar results. ^7^^,^^20^

**Table 1.** MASCO-driven rhythms in wild type mice.

Strain	Sex	Age (wks)	Activity recording	MA dosage (%)	Circadian period (h)										References
					DD					LL					
					Baseline	Dissociation	MA treated		MA post-treatment	Baseline	Dissociation	MA treated		MA post-treatment	
							1st comp	2nd comp				1st comp	2nd comp		
C57BL/6J	NA	12-16	Wheel	0.0075, 0.012, 0.0225 ^a^	23.9	Yes	NA	29	23.8						Coward *et al.*, 2001 ^12^
C57BL/6	♂♀	8-20	Wheel	0.005 → 0.01	23.64	No	24.04 → 24.17		23.74						Tataroglu *et al.*, 2006 ^11^
				0.005 → 0.01	23.75	Yes	24.17 → 23.85	27.50 → 26.96	23.25						
				0.01	23.62	No	24.14		23.71						
				0.01	23.67	Yes	24.04	27.53	23.72						
C57BL/6				0.005						AR (112-1976 lux)	No	27.44			
										26.29 (112-1976 lux)	No	28.21			
C57BL/6 (SCNx)				0.005	AR	No	26.29		AR or 23.54						
C57BL/6J	♀	12	Wheel	0.0025	NA	Yes	24.0-24.4	26.2-36.7	23.4-23.7						Honma *et al.*, 2008 ^14^
C57BL/6J	♂♀	13	Wheel	0.005	NA	No	24.3								Pendergast *et al.*, 2012 ^6^
C57BL/6J	♂♀	6-39	Wheel	0.005	23.63	No	24.23			25.32 (200-300 lux)	No	29.25			Pendergast *et al.*, 2013 ^19^
C57BL/6J (SCNx)					AR	No	29.25, 30.58								
C57BL/6J (PER2::LUC)	NA	4-6	Wheel	0.005	23.67	No	24.67								Salaberry *et al.*, 2017 ^28^
C57BL/6J	♂♀	9-17	IR	0.005	∼24	Yes	∼24	∼28							Ouk *et al.*, 2016 ^31^
		25-33			∼24	Yes	∼24	∼35							
		49-56			∼24	Yes	∼24	∼35							
C57BL/6J	♂	10-15	Wheel	0.005	23.54	No	24.05			26.36 (350 lux)	No	28.31			Mohawk *et al.*, 2009 ^23^
				0.01			25.09					35.09			
CBA x C57BL/6	♂♀	4.8	IR	0.005→0.01	23.7	No	24.2 → 47.3								Cuesta *et al.*, 2012 ^29^
						Yes	24.3 → ?	28.5 → 47.3							
		7.5		0.005	23.7	No	23.9								
						Yes	24	∼30							
CBA x C57BL/6J	♂♀	8	IR	0.005	NA	Yes	∼24	∼25.8							Ouk *et al.*, 2018 ^30^
BALB/c	♀	6-19	Wheel	0.005	23.5	No	24.1		23.8						Masubuchi *et al.*, 2001 ^13^
					23.3	Yes	23.5, 23.9	28.5-33.2	23.8						
C3H (rd +/+)	♂	8-20	Wheel	0.0025 → 0.0065 → 0.01	23.44	No	23.44 → 23.44 → 23.44		23.39						Tataroglu *et al.*, 2006 ^11^

					∼23.61	Yes	23.50→ 23.50→ 23.50	29.50 → 29.00 → 26.83	23.56						


	♀				∼23.30	No	23.47→ 23.53→ 23.70		∼23.40						


C3H (rd +/+)	♂			0.005						AR (112-1976 lux)	No	24.63			
										25.22 (112-1976 lux)	No	∼24.94			
C3H (rd +/+) (SCNx)	♂♀			0.005	AR	No	24.54								

MA: Methamphetamine, DD: Constant darkness, LL: Constant light, AR: arrhythmic, NA: information is not available, IR: Infrared Sensor, SCN-X: SCN-lesioned, →: MA dose increased.

^a^ Amphetamine.

First, we consider MASCO in the absence of the SCN ( Figure 2A). Mice with SCN lesions and no methamphetamine treatment have arrhythmic or ultradian locomotor activity (i.e., there is no apparent MASCO output rhythm, but see later discussion about residual quasi-circadian rhythms in arrhythmic circadian mutant mice). Therefore, we hypothesize that MASCO is a weak oscillator without methamphetamine, so it alone cannot drive an output activity rhythm continuously. Methamphetamine treatment of SCN lesioned mice results in a robust free-running activity rhythm with a long period ranging from 26 h to 30 h. These data suggest that MASCO is a strong oscillator with a long period in the presence of methamphetamine.

Next, we examine the putative relationship between the SCN and MASCO without methamphetamine treatment ( Figure 2B, C). In DD, the SCN is a strong oscillator and MASCO is a weak oscillator, resulting in strong coupling of the SCN to MASCO ( Figure 2B). The period of the activity rhythm, which may be an integration of the output of the coupled SCN and MASCO, is about 23.7 h. In LL, the amplitude of the SCN oscillator is weakened and the period lengthened, but we propose that the SCN oscillator is still stronger than MASCO in LL without methamphetamine treatment ( Figure 2C). The period of the resulting activity rhythm is lengthened in LL, to about 26 h. Since MASCO is a very weak oscillator without methamphetamine treatment, there is only 1 rhythmic component in DD and LL in the absence of methamphetamine treatment.

**Figure 3.**
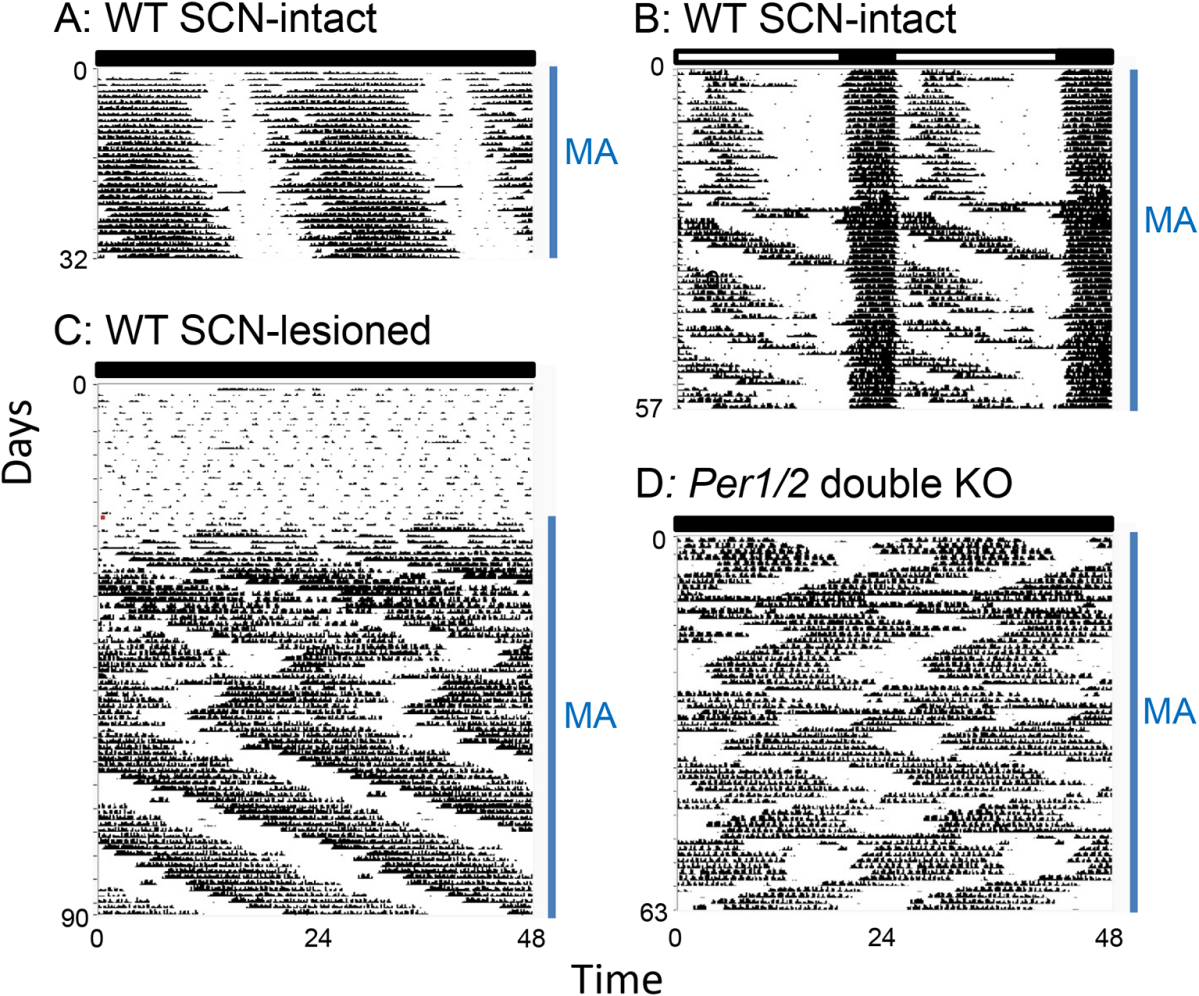
Examples of MASCO-driven activity rhythms in mice. A: Period lengthening of the activity rhythm during short-term methamphetamine administration. The rhythm is controlled by coupled SCN and MASCO in wild-type mice in DD. B: Dissociation of SCN and MASCO-driven activity rhythms by methamphetamine administration in a wild-type mouse in an LD cycle. C: The MASCO-driven activity rhythm in an SCN-lesioned wild-type mouse revealed by methamphetamine administration (NOTE: birhythmicity). D: Birhythmicity of the MASCO-driven activity rhythm in *Per1/2* double knockout mice in DD. MA: methamphetamine administration. The actograms were generated from original data published in Pendergast *et al.*, 2013 ^19^ and 2014. ^57^ The data are re-plotted with the same x-y scale, so the periods of the free-running rhythms can be compared by the angle of the slope. Figure modified from Yamazaki 2019 ^56^ with permission.

Coupling between the SCN and MASCO changes upon methamphetamine administration because methamphetamine treatment increases the amplitude of MASCO ( Figure 2D, E). In DD, the SCN and MASCO are both strong oscillators during treatment with methamphetamine, resulting in strong bi-directional coupling between the two oscillators ( Figure 2D). We propose that MASCO is a stronger oscillator than the SCN in the presence of methamphetamine. Upon methamphetamine treatment, there is one free-running activity rhythm with an ~24 h period ( Figure 3A), which is slightly longer than the period without methamphetamine (23.6 h). However, prolonged methamphetamine administration induces two activity components, one rhythm driven by the SCN and another rhythm driven by MASCO. During this dissociation, the period of the MASCO-driven activity rhythm gets much longer, typically in the 26-30 h range, which approximates the period in SCN-lesioned animals administered methamphetamine. Occasionally, the MASCO-driven activity rhythm exhibits circabidian periods around 48 h during prolonged methamphetamine administration. This dissociation of SCN- and MASCO-driven rhythms can be also seen in LD conditions, suggesting that MASCO is not light-entrainable ( Figure 3B). There is also evidence that MASCO can govern the SCN rhythm. *In vivo* multi-unit activity recordings from the SCN of freely behaving golden hamsters treated with methamphetamine showed that the SCN electrical activity rhythm “free-ran” in the LD cycle with a long period. ^21^ During prolonged methamphetamine treatment the SCN-driven rhythm was no longer observed in LD and DD. These data suggest that with methamphetamine administration, MASCO can govern the SCN and the activity rhythm driven by coupled MASCO-SCN free-runs in LD. MASCO remains a very strong oscillator in LL, but the SCN oscillator amplitude weakens ( Figure 2E). The period of the activity rhythm is longer in LL than in DD because the SCN period is longer and because the contribution of MASCO output to the activity rhythm is greater.

Background strain also affects activity rhythm periods of mice treated with methamphetamine. The Menaker group treated C3H mice with methamphetamine and showed no effect on the period of the activity rhythm (red text in Figure 2). ^11^ They therefore lesioned the SCN in C3H mice to study the MASCO. They found that SCN lesioned C3H mice administered methamphetamine had an activity rhythm period of 24.5 h, which was much shorter than the MASCO-driven rhythm in C57BL/6J mice. Thus, the lack of period changes in C3H mice upon methamphetamine treatment is likely because the period of MASCO is very close to the period of the SCN in this strain. However, they also reported two dissociated activity rhythms (23.5 h and ~29 h) in male C3H mice given a higher methamphetamine dose (0.0065–0.01%). This suggests that the period of MASCO in C3H mice can get longer during prolonged, higher doses of methamphetamine administration. The effects of prolonged methamphetamine administration and the influence of genes on the period of MASCO are further discussed in the next section.

## Canonical circadian genes are not essential for MASCO

The next surprising discovery about MASCO was that it can oscillate without the canonical circadian genes that are essential for rhythmicity in nearly all circadian oscillators. These studies were performed in circadian mutant mice in DD so the mice had no circadian activity rhythms ( Table 2; most mutants exhibited ultradian rhythms). The first study was in arrhythmic *Clock ^Δ19^* mutant mice. Two independent laboratories showed that *Clock ^Δ19^* mutant mice expressed MASCO-driven activity rhythms upon amphetamine or methamphetamine administration. ^12^^,^^22^ Similar results were later found in arrhythmic *Cry1/2* double knockout mice, *Bmal1* knockout mice, *Per1/2* double knockout mice, and *Per1/2/3* triple knockout mice. ^6^^,^^14^^,^^15^^,^^17^ Collectively these studies demonstrate that the canonical circadian genes, which are essential for timekeeping in nearly all circadian clocks, are not required for MASCO to oscillate.

**Table 2.** MASCO-driven rhythms in circadian gene mutant and engineered mice.

Gene disrupted	Line	Strain	Sex	Age (wks)	Activity recording	MA dosage (%)	Circadian period (h)										References
							DD					LL					
							Baseline	Dissociation	MA treated		MA post-treatment	Baseline	Dissociation	MA treated		MA post-treatment	
									1st component	2nd component				1st component	2nd component		
*Clock*	*Δ19* mutant	C57BL/6J	NA	12-16	Wheel	0.0075, 0.012, 0.0225 ^a^	AR	No	∼29		AR or 28.5						Coward *et al*., 2001 ^12^
*Clock*	*Δ19* mutant	BALB/c	♀	6-19	Wheel	0.005	AR	No	27.5-42.9		26.8-29.3						Masubuchi *et al*., 2001 ^13^
*Clock*	*Δ19* mutant	C57BL/6	NA	NA	Wheel	0.005	27.83	No	28.5								Mohawk *et al*., 2009 ^15^
*Cry1 and Cry2*	Global KO	C57BL/6	♀	12	Wheel	0.0025	AR	No	20.8-32.3		AR or 22.3						Honma *et al*., 2008 ^14^
*Cry1 and Cry2*	Global KO	C57BL/6	NA	NA	Wheel	0.005	AR	No	24.50-35.33								Mohawk *et al*., 2009 ^15^
*Cry1 and Cry2* (SCN-X)						0.005	AR		26.67								
*Bmal1*	Global KO	C57BL/6	NA	NA	IR	0.0035 → 0.005	AR	No	32.77								Mohawk * et al.*, 2009 ^15^
*Bmal1*	Global KO	C57BL/6J	NA	NA	Wheel	0.00125 → 0.0025 → 0.005 → 0.01	AR (~4)	No	~6 → ~9 → ~14 → ~20								Blum *et al*., 2014 ^17^
						0.01 ^**a**^	AR (~3)		∼8								
*Npas2*	Global KO	C57BL/6	NA	NA	Wheel	0.005	23.54	No	24.13								Mohawk * et al.*, 2009 ^15^
*Npas2* (SCN-X)							AR	No	28.17								
*Per1 and Per2*	Global KO	Mixed	NA	NA	Wheel	0.005	AR	No	27.14								Mohawk * et al.*, 2009 ^15^
*Per1, Per2, Per3*	Global KO	C57BL/6J	♂♀	15	Wheel	0.005	AR	No	21.5								Pendergast *et al*., 2012 ^6^
*Per1*	Global KO	C57BL/6J	♂♀	6-39	Wheel	0.005	23.49	No	24.71			27.25 (200-300 lux)	No	34.96			Pendergast *et al*., 2013 ^19^
*Per1* (SCN-X)							AR		29.67								
*Per2*							23.48	No	23.56			23.40 (200-300 lux)	Yes	23.14	30.03		
*Per2* (SCN-X)							AR		27.75								
*Per3*							23.51	No	24.26			25.00 (200-300 lux)	No	30.7			
*Per3* (SCN-X)							AR		26.28								
*Per1 and Per2*							AR	No	23.08 (17.00-32.50)			AR (200-300 lux)	No	17.58, 17.17, 35.33			
*Per2 and Per3*							23.12	No	23.28								
*Rev Erbα*	Global KO with PER2::LUC	C57BL/6J	NA	4-6	IR	0.005	23.36	No	23.25								Salaberry *et al*., 2017 ^28^
*CK1ε*	*Tau* KI	C57BL/6	NA	NA	wheel	0.005	20.11	Yes	NA	25.86							Mohawk *et al*., 2009 ^15^
*CK1ε* (SCN-X)							AR	No	34.09								
*Human Huntington Disease gene*	*R6/2* TG	CBAxC57BL/6	♂♀	4.8	IR	0.005 → 0 01	23.7	No	23.7 → 23.1								Cuesta *et al*., 2012 ^29^
								Yes	23.7	29.4 → 23.1							
				7.5		0.005	∼23.4	No	23.3 → 23.3								
*Human Huntington Disease gene*	*R6/2 TG*	CBAxC57BL/6	♂♀	6	IR	0.005	∼23.6	No	∼23.6								Ouk *et al*., 2018 ^30^
*Human Huntington Disease gene*	*Q175* TG	C57BL/6J	♂♀	9-17	IR	0.005	∼24	Yes	∼24	∼40							Ouk *et al*., 2016 ^31^
				25-33				No	∼24								
				49-56				No	∼24								
*Gsk3β*	Global KO (Heterozygous)	C57BL/6	♂♀	NA	Wheel	0.005	∼23.7	No	∼27.0								Mohawk *et al*., 2009 ^23^

MA: Methamphetamine, DD: Constant darkness, LL: Constant light, AR: arrhythmic, NA: information is not available, IR: Infrared Sensor, SCN-X: SCN-lesioned, →: MA dose increased.

^a^ Amphetamine.

MASCO-driven activity rhythms in single circadian gene knockout mice have also been studied ( Table 2). In these studies, the mice were SCN lesioned to remove the influence of the SCN (since we predict it is coupled to MASCO, see Figure 2) and the autonomous period of MASCO was measured. SCN-lesioned *Npas2* knockout mice, as well as single *Per1*, *Per2*, or *Per3* knockout mice had activity rhythms with 26–30 h periods when treated with methamphetamine ( Table 2). The MASCO-driven behavior periods in these single mutant mice on the C57BL/6J background were similar to those observed in SCN-lesioned WT C57BL/6J mice, demonstrating that single gene knockouts do not have a discernible effect on the period of MASCO. Homozygous *tau* mutant mice exhibited a shortened free-running period (~20 h) in DD. SCN-lesioned homozygous *tau* mutant mice were arrhythmic in DD and methamphetamine administration revealed a MASCO-driven activity rhythm with a ~34 h period. These data showing that the *tau* mutation does not influence the period of MASCO in the same way it does the SCN period, suggests that the molecular timekeeping mechanism in MASCO is different from that in canonical circadian oscillators. ^15^ It was also shown that methamphetamine induced a very long free-running period (~27 h) in heterozygous *Gsk3* *β *mutant mice in DD even though the mutation did not affect the circadian period in DD without methamphetamine). ^23^ These data suggest that *Gsk3* *β *mutant mice have increased sensitivity to methamphetamine.

Note that several studies have also been performed in single circadian gene mutant mice with intact SCN. These studies are difficult to interpret because the resulting locomotor activity is driven by coupled SCN and MASCO, and the mutation could affect one or both of these oscillators. For these reasons, studying the effects of single gene knockouts on MASCO during short-term methamphetamine administration must be performed in SCN lesioned animals (except *Bmal1* KO mice with disabled circadian clocks, see Table 2). Alternatively, prolonged methamphetamine administration can reveal two independent free-running components – one from the SCN and another from MASCO. However, experiments designed to observe free-running periods of both rhythms with chronic methamphetamine treatment should be performed in DD.

## Sudden period changes are a unique rhythmic property of MASCO

MASCO-driven behavior rhythms in SCN-lesioned animals or in animals with genetically disabled SCN rhythmicity, where rhythms are solely driven by MASCO, are typically less stable than the rhythms of mice with intact SCN, where the rhythms are driven by coupled MASCO-SCN. Sudden period changes in the MASCO-driven circadian behavior rhythms in mice without functional SCN are often recorded. In some publications, the authors described the details of these spontaneous period changes (e.g., occurred after a cage change). ^14^^,^^19^^,^^22^ But in most studies, the authors just reported the unstable nature of MASCO rhythmicity. In general, the MASCO-driven circadian rhythm is ~26–30 h in DD (occasionally rhythms with periods twice as long as a day, circabidian or much longer, infradian) were reported during prolonged methamphetamine administration, see below). Studies frequently report a zigzag pattern of activity ( Figure 3C, D), where a long-period rhythm suddenly shifts to a short-period rhythm and then suddenly switches back to a longer period rhythm. We have observed this zigzag pattern in *Per1/2* double knockout mice and SCN-lesioned wild type mice. ^19^ We noticed that the period changes often coincided with the days we opened the light-tight boxes for visual inspection (note that we used an infrared viewer so mice were not exposed to visible light). ^19^ This zigzag pattern can be interpreted as either two weakly coupled oscillators exhibiting relative coordination or a single oscillator showing birhythmicity, which is the two stable regimes of a limit cycle oscillator. ^24^ Regardless of the underlying mechanism, this may be a unique characteristic of MASCO.

The period of the MASCO-driven behavior rhythm is also variable and generally becomes longer over the course of methamphetamine administration. Studies of prolonged methamphetamine administration to arrhythmic mutant mice and SCN-lesioned animals often report that the period of MASCO-driven behavior rhythm is circabidian or even much longer, infradian (one report showed that the period of the MASCO-driven activity rhythm reached ~100 h). ^14^^,^^15^^,^^17^^,^^22^ In addition to period instability, a characteristic of MASCO is that its output rhythms often exceed the circadian range.

## Genetic factors that influence the period of MASCO

Although most circadian mutations and genetic modification of circadian genes have no noticeable effects on MASCO, several genetic factors do affect MASCO. First, the period of MASCO differs by mouse strain. In C57BL/6J mice, methamphetamine treatment reveals an activity rhythm with a period much longer (26–30 h) than the SCN-controlled rhythm. In contrast, the period of the activity rhythm in C3H mice treated with methamphetamine is indistinguishable from the period of the rhythm without methamphetamine. There are three possible reasons that methamphetamine has no apparent effect on activity rhythms in C3H mice: (i) The animal is insensitive to methamphetamine; (ii) The animal has a MASCO with a short period, or; (iii) The animal does not have a functional MASCO. Studies in C3H mice, as we discussed previously, showed that they have a functional MASCO with a short period that is sensitive to methamphetamine. However, the genetics that cause the short period of MASCO are not known yet.

Second, MASCO-driven rhythms are altered in some circadian mutant mice. Methamphetamine administration to *Per2* knockout mice does not affect the period of the free-running rhythm in DD. A confusing result is that methamphetamine revealed a typical long period of MASCO in SCN-lesioned *Per2* knockout mice. This excludes all three possible mechanisms described above. When methamphetamine was given to SCN-intact *Per2* knockout mice in LL, two different free-running periods appeared, one was ~23 h and the other was ~30 h. It is known that LL does not affect the period of *Per2* knockout mice (they lack a parametric light effect). ^25^^–^^27^ Therefore, in LL, it is likely that the short period rhythm is driven by the SCN and the long period rhythm is driven by MASCO. In the case of *Per2* knockout mice, it is likely that coupling between the SCN and MASCO is affected. To explain the MASCO rhythms in DD, we concluded that the MASCO driven rhythm was masked and therefore not expressed in the behavior rhythm. Methamphetamine also does not affect the periods of the MASCO-driven rhythms in *Rev-Erb *α** knockout mice and in R6/2 and Q175 Huntington’s disease model mice. ^28^^–^^31^ Because SCN lesions have not yet been performed in those mice, the cause of methamphetamine insensitivity is unknown.

Third, some gene mutants affect the period of MASCO. *Per1/2/3* triple knockout mice express a MASCO-driven circadian rhythm that has an ~20 h period. None of the SCN-lesioned single *Per* gene knockout mice ( *Per1* or *Per2* or *Per3*) exhibited short-period MASCO-driven rhythms. *Per1/2* double knockout mice exhibited activity rhythms that alternate between short and long periods. This zigzag pattern is commonly seen in the MASCO driven rhythm, so it is tempting to speculate that the period tends to be stable at the long period in most mutants, but at the short period in *Per1/2/3* triple knockout mice, so *Per1/2/3* triple knockout mice stably exhibit the shorter MASCO period.

## Sex differences in MASCO-driven activity

Studies of MASCO have been performed in both male and female rodents ( Table 1). Honma’s group tested for sex differences in the response of activity rhythms to methamphetamine treatment. They housed rats in the LD cycle and administered methamphetamine. They found that the MASCO-driven activity rhythm typically dissociated from the SCN-driven rhythm in female rats, but they observed this dissociation less frequently in male rats. ^8^ Therefore, Honma’s group used females for their MASCO studies. On the other hand, Menaker’s group observed period elongation by methamphetamine in both male and female mice in DD. ^11^ They and others also observed that methamphetamine revealed MASCO-driven activity rhythms in both male and female SCN lesioned mice ( Tables 1 and 2). ^11^ Therefore, it is likely that MASCO is present in both sexes but coupling between the SCN and MASCO may be weaker in females, which is why the MASCO-driven rhythm dissociates more readily in females.

## Role of dopamine in MASCO

A primary action of methamphetamine in the brain is to elevate extracellular monoamine neurotransmitters, dopamine, serotonin and norepinephrine, in synapses. Several studies have shown that using pharmacological or genetic models to change dopamine levels or signalling affects MASCO rhythms.

The pharmacological approach has been to treat animals with haloperidol which is a non-selective dopamine D2 receptor antagonist. Honma’s group demonstrated that injection of haloperidol produced phase-dependent shifts in MASCO-driven behavior rhythms in SCN-lesioned rats. ^32^ Intramuscular injection of haloperidol offset the dissociated MASCO-driven activity component in rats. ^33^ Chronically treating C57BL/6 wild-type and *Bmal1* knockout mice with haloperidol shortened the period of the MASCO-driven activity rhythm. ^17^ On the other hand, one study showed that haloperidol did not affect MASCO period in SCN-intact wild-type mice (CBA x C57BL/6J). ^30^

MASCO has also been studied in genetically modified mice with altered dopamine signalling. Sodium-dependent dopamine transporter ( *Slc6a3*) knockout mice exhibited two activity components (~23.5 h and ~27 h) in DD without methamphetamine. ^17^ R6/2 Huntington’s disease model mice have compromised dopamine signalling and MASCO-driven activity rhythms. However, when these mice were treated with L-dopa, the MASCO-driven activity rhythm was rescued. ^29^ Together these studies suggest that the MASCO-driven circadian rhythm is revealed by increased dopamine signalling. However, it is possible that the monoamine serotonin also affects MASCO. The serotonin-specific reuptake inhibitor, paroxetine, is often used as an antidepressant in Huntington’s disease and also rescues the methamphetamine-insensitive phenotype in the R6/2 mouse model. ^30^ Studying the MASCO-driven circadian behavior rhythm in dopamine receptor knockout mice will likely provide useful information about the specific role of dopamine in MASCO.

## Is MASCO an autonomous circadian oscillator in normal physiological conditions?

In the presence of methamphetamine, MASCO is a robust circadian oscillator. During prolonged chronic methamphetamine administration, MASCO can take over SCN function and control the locomotor activity rhythm. In SCN-lesioned animals, MASCO can at least partially substitute for the SCN and coordinate the phases of oscillators in peripheral tissues. ^34^ MASCO also can drive core body temperature, feeding, drinking, and plasma corticosterone rhythms in SCN-lesioned rats. ^35^ However, we do not know the nature of MASCO in the absence of methamphetamine treatment. It is possible that MASCO is a weak circadian oscillator and strongly coupled to the SCN, therefore we cannot observe the MASCO-driven rhythm in the absence of methamphetamine. When methamphetamine is administered, MASCO becomes a strong oscillator and eventually takes over SCN function. It is also possible that MASCO is not a circadian oscillator in the absence of methamphetamine. In the past, researchers have proposed that MASCO is a dopamine ultradian oscillator whose period is elongated by methamphetamine. ^17^^,^^36^ It is also possible that MASCO is not any kind of oscillator without methamphetamine.

The anatomical location of MASCO has not yet been identified so it is not possible to collect biochemical and molecular measurements to determine whether MASCO is rhythmic in normal conditions (i.e., in the absence of methamphetamine). However, there are several studies that measured circadian rhythms in physiology and gene expression in animals exhibiting SCN-MASCO dissociation. The melatonin rhythm in rats aligns with the SCN activity rhythm, but not with the MASCO-driven rhythm. ^13^ Extra-SCN brain areas, including the caudate putamen and parietal cortex, oscillate in phase with the MASCO-driven activity rhythm. ^13^ These data indicate that the SCN and MASCO govern distinct physiological rhythms. We also recently discovered that *Per1/2/3* triple knockout mice spontaneously express an ~20 h quasi-circadian rhythm every 20 days in DD (in normal conditions without methamphetamine). ^37^ The period of MASCO in *Per1/2/3* mice is ~20 h, which matches the period of this spontaneous rhythm. Other arousal stimuli, including running wheels and palatable meals, also reveal MASCO-like behavior rhythms in *Per1/2/3* triple knockout mice. ^38^ Thus, there is evidence to suggest that MASCO is continuously ticking with a circadian period even in normal conditions. We can confidently say that MASCO is an oscillator that can control physiological rhythms in the presence of arousing stimuli or drugs that increase dopamine signalling.

## Proposed functional significance of MASCO

Thus far we have described the properties of MASCO revealed by treatment of rodents with stimulants. However, this is a very artificial situation that is rarely encountered under natural or physiological conditions. Thus, a critical question remains: what is the function of MASCO? First, we will review the experimental results that provide hints about MASCO function, and then we propose a working model of MASCO’s function and how it interacts with the circadian system.

The roles of the SCN are well-studied, so the approach to studying MASCO has been to investigate SCN-controlled functions in the presence of a robust MASCO oscillator (i.e., with methamphetamine in either SCN intact or SCN lesioned rodents). One major function of the SCN is to orchestrate the phase of peripheral circadian oscillators. ^39^^–^^41^ The Menaker group showed that MASCO can at least partially substitute for this SCN function. ^34^ They showed that the phases of peripheral tissues were coordinated with the phase of the MASCO-driven rhythm in SCN-lesioned mice treated with methamphetamine. It is possible that MASCO controls the rhythm of food intake, which in turn entrains the phases of peripheral oscillators. Nevertheless, this experiment shows that SCN and MASCO can share some functions, by acting at the top of the circadian hierarchy. On the other hand, there are several examples suggesting that the SCN and MASCO have distinct functions. Honma’s group showed that the peak of the melatonin rhythm in rats is always in phase with the SCN, even during SCN-MASCO dissociation, suggesting minimal influence of MASCO on the melatonin rhythm. ^13^ They also showed that the phases of circadian gene expression rhythms in various brain regions coordinate with the phase of the MASCO-driven activity rhythm. ^13^^,^^16^ They also examined circadian gene expression in different brain regions in rats during temporally restricted methamphetamine water availability. ^42^ These data showed that gene expression in dopaminergic brain areas were in phase with MASCO.

In the 1970’s, Aschoff and Wever conducted studies in human subjects at the isolation facility in Andechs, Germany. ^43^ When human subjects were isolated from natural and social environmental cues, their circadian rhythms free-ran with approximate 25-h periods. During long-term isolation, investigators often observed a phenomenon called internal desynchronization. When internal desynchronization occurs, the periods of core body temperature and melatonin rhythms become slightly shorter than before internal desynchronization. At the same time, the period of the sleep-wake cycle typically becomes extremely long (~30 h; but occasionally very short, ~17 h). The researchers concluded that there are two circadian oscillators in humans, one that controls physiology (core body temperature and melatonin) with a period close to 24 h and another that controls the sleep-wake cycle with periods that significantly deviate from 24 h (either extremely longer or shorter than 24 h). This internal desynchronization in humans is very similar to SCN-MASCO dissociation observed in rodents. ^13^^,^^29^ In addition, the rapid shifts in the period of the sleep-wake cycle to be either extremely short or long during internal desynchronization are similar to birhythmicity observed with MASCO in rodents. Of note, the interpretation of internal desynchrony data may be different if napping is considered as sleep. Zulley and Campbell reanalyzed the internal desynchronization data of 6 subjects for whom naptime data was available (naps typically occurred between the sleep bouts) and reported that the phase relationship between the core body temperature rhythms and sleep/naps was maintained. As a result of this reanalysis, they concluded that internal desynchronization was between the “psychological day and sleep, but not between biological day and sleep”. ^44^ However, if altered time perception (i.e., psychological day) were the cause of internal desynchronization, then there would be a dissociated sleep period of ~ 48 h, but not periods of 17 h or 30 h that were observed. Thus, even when taking napping into account, the rhythms in isolated human subjects still approximate the dissociated MASCO rhythms in rodents.

The mechanisms directly controlling the human sleep-wake cycle (process S) are still under debate and may or may not require a self-sustained circadian oscillator. ^45^ Researchers have theoretically shown that process S can be explained as an hourglass coupled with a circadian oscillator (process C). However, the observation that features of the sleep-wake cycle during internal desynchronization parallel MASCO studies in rodents support our working hypothesis that the functional significance of MASCO is to control the sleep-wake cycle. More support for a role of MASCO in controlling sleep-wake cycles comes from Honma’s group. ^46^ They showed that a single meal can entrain the sleep-wake rhythm, but not core body temperature or melatonin rhythms in human subjects with internal desynchronization. This is similar to the observation in rodents that MASCO can entrain to restricted feeding. Rietveld and colleagues suggested that the MASCO-driven activity rhythm in SCN-lesioned rats can be simulated by the hourglass model and pointed out similarities between the MASCO-driven activity rhythm in rats and human sleep regulation (hourglass model). ^36^ Regardless of whether MASCO is a self-sustained oscillator or an hourglass oscillator, it shares similarities with human sleep cycles.

## Possible roles for MASCO in human disorders

Non-24-Hour Sleep-Wake Rhythm Disorder (N24SWD) is a human circadian disorder where sleep onset moves to a later hour each day. In rare cases, sleep onset moves earlier in persons with N24SWD. ^47^ Recently, the period of the circadian rhythm in a person with N24SWD was measured in forced desynchrony and constant routine conditions. During forced desynchrony, the subject is forced to sleep with a period outside of the circadian range of entrainment (28 h is commonly used). This is used to estimate the period of the circadian pacemaker (presumably the SCN) by measuring the phase difference between melatonin onsets and the rectal temperature rhythm under constant conditions at the beginning and end of forced desynchrony. Czeisler and colleagues surprisingly found that the circadian pacemaker period in the individual with N24SWD was within the range of normal circadian periods (24.5 h). ^48^ This individual exhibited ~25 h self-selected sleep cycles for a few weeks before and after forced desynchrony. This raises the possibility that N24SWD is internal desynchronization that occurs in the normal natural/social environment.

Methylphenidate is a common treatment for people with attention-deficit/hyperactivity disorder (ADHD). Methylphenidate increases dopamine at synapses by blocking dopamine reuptake transporters and by increasing the expression of dopamine transporters in the brain. In rodents, methylphenidate treatment induces SCN-MASCO dissociation. ^20^ It is estimated that 25–50% of people with ADHD experiences sleep problems ^49^^,^^50^ and there is a positive correlation between the dose of methylphenidate and sleep problems in children with ADHD. ^51^ It is also commonly reported that patients with schizophrenia experience disordered sleep, including phase-advanced/delayed or non-24 h sleep. ^52^ Although the sleep irregularities could be caused by anti-psychotic medications, the non-24 h sleep phenotype is similar to MASCO-driven behavior in rodents. Blum and colleagues also described similarities between DUO and sleep/activity patterns in bipolar patients. ^17^

## Conclusions

It is now well established that disruption of circadian rhythms and impaired sleep increase the risk of human diseases. Sleep and circadian rhythms are impaired in several neurological disorders and in persons with drug addiction. ^53^^–^^55^ However, we know little about the mechanisms of this sleep disruption. MASCO is an understudied circadian oscillator, but its link to dopaminergic signalling and rewarding stimuli is clear. We are learning more about how MASCO is altered in mutant mice, and we see parallels between sleep in human disorders and MASCO rhythms in rodents. Thus, increasing our knowledge of how MASCO regulates physiological and behavioral rhythms could help us understand how to manage circadian rhythm and sleep disruption in humans. ^17^

## Data availability

No data are associated with this article.

## References

[1] Reppert SM Weaver DR : Coordination of circadian timing in mammals. *Nature.* 2002;418(6901):935–941. 10.1038/nature00965 12198538

[2] Takahashi JS : Transcriptional architecture of the mammalian circadian clock. *Nat. Rev. Genet.* 2017;18(3):164–179. 10.1038/nrg.2016.150 27990019 PMC5501165

[3] Koronowski KB Sassone-Corsi P : Communicating clocks shape circadian homeostasis. *Science.* 2021;371(6530): eabd0951. 33574181 10.1126/science.abd0951 PMC8123919

[4] Green CB Takahashi JS Bass J : The meter of metabolism. *Cell.* 2008;134(5):728–742. 10.1016/j.cell.2008.08.022 18775307 PMC3760165

[5] Pendergast JS Yamazaki S : Extra-SCN circadian pacemakers. *Biological Clocks.* Honma K Honma S , editors. Sapporo: Hokkaido Univ. Press;2017; p.141–152.

[6] Pendergast JS : Period determination in the food-entrainable and methamphetamine-sensitive circadian oscillator(s). *Proc. Natl. Acad. Sci. U. S. A.* 2012;109(35):14218–14223. 10.1073/pnas.1206213109 22891330 PMC3435193

[7] Ikeda Y Chiba Y : Effects of Psychotropics on Circadian Motor Activity in Rats. *Toward chronopharmacology: proceedings of a satellite symposium to the 8th International Congress of Pharmacology, Nagasaki, Japan, 27-28 July 1981.* Takahashi R Halberg F Walker CA , editors. Pergamon Press;1982; pp.3–10.

[8] Honma K Honma S Hiroshige T : Disorganization of the rat activity rhythm by chronic treatment with methamphetamine. *Physiol. Behav.* 1986;38:687–695. 10.1016/0031-9384(86)90265-9 3823184

[9] Honma K Honma S Hiroshige T : Activity rhythms in the circadian domain appear in suprachiasmatic nuclei lesioned rats given methamphetamine. *Physiol. Behav.* 1987;40(6):767–774. 10.1016/0031-9384(87)90281-2 3313452

[10] Honma S Honma K Hiroshige T : Methamphetamine induced locomotor rhythm entrains to restricted daily feeding in SCN lesioned rats. *Physiol. Behav.* 1989;45(5):1057–1065. 10.1016/0031-9384(89)90237-0 2780867

[11] Tataroglu O : The methamphetamine-sensitive circadian oscillator (MASCO) in mice. *J. Biol. Rhythm.* 2006;21(3):185–194. 10.1177/0748730406287529 16731658

[12] Coward DJ Cain SW Ralph MR : A circadian rhythm in mice that is unaffected by the period mutation at clock. *Biol. Rhythm. Res.* 2001;32(2):233–242. 10.1076/brhm.32.2.233.1356

[13] Masubuchi S : Clock genes outside the suprachiasmatic nucleus involved in manifestation of locomotor activity rhythm in rats. *Eur. J. Neurosci.* 2000;12(12):4206–4214. 11122332

[14] Honma S : Circadian behavioral rhythms in Cry1/Cry2 double-deficient mice induced by methamphetamine. *J. Biol. Rhythm.* 2008;23(1):91–94. 10.1177/0748730407311124 18258761

[15] Mohawk JA Baer ML Menaker M : The methamphetamine-sensitive circadian oscillator does not employ canonical clock genes. *Proc. Natl. Acad. Sci. U. S. A.* 2009;106(9):3519–3524. 10.1073/pnas.0813366106 19204282 PMC2651344

[16] Natsubori A Honma K Honma S : Differential responses of circadian Per2 rhythms in cultured slices of discrete brain areas from rats showing internal desynchronisation by methamphetamine. *Eur. J. Neurosci.* 2013;38(4):2566–2571. 10.1111/ejn.12265 23725367

[17] Blum ID : A highly tunable dopaminergic oscillator generates ultradian rhythms of behavioral arousal. *elife.* 2014;3:e05105. 10.7554/eLife.05105 25546305 PMC4337656

[18] Ohta H Yamazaki S McMahon DG : Constant light desynchronizes mammalian clock neurons. *Nat. Neurosci.* 2005;8(3):267–269. 10.1038/nn1395 15746913

[19] Pendergast JS Niswender KD Yamazaki S : The complex relationship between the light-entrainable and methamphetamine-sensitive circadian oscillators: evidence from behavioral studies of Period-mutant mice. *Eur. J. Neurosci.* 2013;38:3044–3053. 10.1111/ejn.12309 23869717 PMC3899104

[20] Honma S Honma K : Locomotor rhythms induced by methylphenidate in suprachiasmatic nuclei-lesioned rats. *Neurosci. Lett.* 1992;137(1):24–28. 10.1016/0304-3940(92)90289-J 1625812

[21] Omata K Kawamura H : Effects of methamphetamine upon circadian rhythms in multiple unit activity inside and outside the suprachiasmatic nucleus in the golden hamster (Mesocricetus auratus). *Neurosci. Lett.* 1988;95(1-3):218–222. 10.1016/0304-3940(88)90660-X 3067126

[22] Masubuchi S : Circadian activity rhythm in methamphetamine-treated Clock mutant mice. *Eur. J. Neurosci.* 2001;14(7):1177–1180. 10.1046/j.0953-816x.2001.01749.x 11683910

[23] Mohawk JA : Lithium and genetic inhibition of GSK3beta enhance the effect of methamphetamine on circadian rhythms in the mouse. *Behav. Pharmacol.* 2009;20(2):174–183. 10.1097/FBP.0b013e32832a8f43 19339873 PMC2893036

[24] Decroly O Goldbeter A : Birhythmicity, chaos, and other patterns of temporal self-organization in a multiply regulated biochemical system. *Proc. Natl. Acad. Sci. U. S. A.* 1982;79(22):6917–6921. 10.1073/pnas.79.22.6917 6960354 PMC347245

[25] Pendergast JS Friday RC Yamazaki S : Photic Entrainment of Period Mutant Mice is Predicted from Their Phase Response Curves. *J. Neurosci.* 2010;30(36):12179–12184. 10.1523/JNEUROSCI.2607-10.2010 20826680 PMC2943870

[26] Tamiya H : Rigid Cooperation of Per1 and Per2 proteins. *Sci. Rep.* 2016;6:32769. 10.1038/srep32769 27609640 PMC5016722

[27] Spoelstra K Daan S : Effects of constant light on circadian rhythmicity in mice lacking functional cry genes: dissimilar from per mutants. *J. Comp. Physiol. A Neuroethol. Sens. Neural Behav. Physiol.* 2008;194(3):235–242. 10.1007/s00359-007-0301-3 18057941

[28] Salaberry NL Mateo M Mendoza J : The Clock Gene Rev-Erbalpha Regulates Methamphetamine Actions on Circadian Timekeeping in the Mouse Brain. *Mol. Neurobiol.* 2017;54(7):5327–5334. 10.1007/s12035-016-0076-z 27581301

[29] Cuesta M Aungier J Morton AJ : The methamphetamine-sensitive circadian oscillator is dysfunctional in a transgenic mouse model of Huntington's disease. *Neurobiol. Dis.* 2012;45(1):145–155. 10.1016/j.nbd.2011.07.016 21820053

[30] Ouk K : Chronic paroxetine treatment prevents disruption of methamphetamine-sensitive circadian oscillator in a transgenic mouse model of Huntington's disease. *Neuropharmacology.* 2018;131:337–350. 10.1016/j.neuropharm.2017.12.033 29274752

[31] Ouk K Aungier J Morton AJ : Progressive gene dose-dependent disruption of the methamphetamine-sensitive circadian oscillator-driven rhythms in a knock-in mouse model of Huntington's disease. *Exp. Neurol.* 2016;286:69–82. 10.1016/j.expneurol.2016.09.007 27646506

[32] Honma S Honma K : Phase-dependent phase shift of methamphetamine-induced circadian rhythm by haloperidol in SCN-lesioned rats. *Brain Res.* 1995;674(2):283–290. 10.1016/0006-8993(95)00027-N 7796108

[33] Rietveld WJ : The circadian control of behavior in the rat affected by the chronic application of methamphetamine. *Prog. Clin. Biol. Res.* 1987;227B:513–517. 3628359

[34] Pezuk P : Circadian organization is governed by extra-SCN pacemakers. *J. Biol. Rhythm.* 2010;25(6):432–441. 10.1177/0748730410385204 21135159

[35] Honma S : Rhythms in behaviors, body temperature and plasma corticosterone in SCN lesioned rats given methamphetamine. *Physiol. Behav.* 1988;44(2):247–255. 10.1016/0031-9384(88)90146-1 3237831

[36] Ruis JF : Effects of T cycles of light/darkness and periodic forced activity on methamphetamine-induced rhythms in intact and SCN-lesioned rats: explanation by an hourglass-clock model. *Physiol. Behav.* 1990;47(5):917–929. 10.1016/0031-9384(90)90020-5 2388949

[37] Morris M Yamazaki S Stefanovska A : Multiscale Time-resolved Analysis Reveals Remaining Behavioral Rhythms in Mice Without Canonical Circadian Clocks. *J. Biol. Rhythm.* 2022;37(3):310–328. 10.1177/07487304221087065 35575430 PMC9160956

[38] Flores DE Bettilyon CN Yamazaki S : Period-independent novel circadian oscillators revealed by timed exercise and palatable meals. *Sci. Rep.* 2016;6:21945. Reference Source 26904978 10.1038/srep21945 PMC4764932

[39] Davidson AJ Yamazaki S Menaker M : SCN: ringmaster of the circadian circus or conductor of the circadian orchestra? *Novartis Found. Symp.* ,2003;253:110–121. discussion 121–125, 281–284. 14712917

[40] Yoo SH : PERIOD2::LUCIFERASE real-time reporting of circadian dynamics reveals persistent circadian oscillations in mouse peripheral tissues. *Proc. Natl. Acad. Sci. U. S. A.* 2004;101(15):5339–5346. 10.1073/pnas.0308709101 14963227 PMC397382

[41] Yamazaki S : Resetting central and peripheral circadian oscillators in transgenic rats. *Science.* 2000;288(5466):682–685. 10.1126/science.288.5466.682 10784453

[42] Natsubori A Honma K Honma S : Dual regulation of clock gene Per2 expression in discrete brain areas by the circadian pacemaker and methamphetamine-induced oscillator in rats. *Eur. J. Neurosci.* 2014;39(2):229–240. 10.1111/ejn.12400 24438490

[43] Wever RA : *The Circadian System of Man; Results of Experiments Under Temporal Isolation.* Springer-Verlag;1979;276.

[44] Zulley J Campbell SS : Napping behavior during “spontaneous internal desynchronization”: sleep remains in synchrony with body temperature. *Hum. Neurobiol.* 1985;4(1):123–126. 4030422

[45] Borbely A : The two-process model of sleep regulation: Beginnings and outlook. *J. Sleep Res.* 2022;31(4):e13598. 10.1111/jsr.13598 35502706 PMC9540767

[46] Yamanaka Y : A fixed single meal in the subjective day prevents free-running of the human sleep-wake cycle but not of the circadian pacemaker under temporal isolation. *Am. J. Physiol. Regul. Integr. Comp. Physiol.* 2022;323(1):R16–R27. 10.1152/ajpregu.00262.2021 35470708

[47] Auger RR : Clinical Practice Guideline for the Treatment of Intrinsic Circadian Rhythm Sleep-Wake Disorders: Advanced Sleep-Wake Phase Disorder (ASWPD), Delayed Sleep-Wake Phase Disorder (DSWPD), Non-24-Hour Sleep-Wake Rhythm Disorder (N24SWD), and Irregular Sleep-Wake Rhythm Disorder (ISWRD). An Update for 2015: An American Academy of Sleep Medicine Clinical Practice Guideline. *J. Clin. Sleep Med.* 2015;11(10):1199–1236. 10.5664/jcsm.5100 26414986 PMC4582061

[48] Emens JS : Behaviorally and environmentally induced non-24-hour sleep-wake rhythm disorder in sighted patients. *J. Clin. Sleep Med.* 2022;18(2):453–459. 10.5664/jcsm.9612 34402783 PMC8805008

[49] Sung V : Sleep problems in children with attention-deficit/hyperactivity disorder: prevalence and the effect on the child and family. *Arch. Pediatr. Adolesc. Med.* 2008;162(4):336–342. 10.1001/archpedi.162.4.336 18391142

[50] Wynchank D : Adult Attention-Deficit/Hyperactivity Disorder (ADHD) and Insomnia: an Update of the Literature. *Curr. Psychiatry Rep.* 2017;19(12):98. 10.1007/s11920-017-0860-0 29086065

[51] Becker SP Froehlich TE Epstein JN : Effects of Methylphenidate on Sleep Functioning in Children with Attention-Deficit/Hyperactivity Disorder. *J. Dev. Behav. Pediatr.* 2016;37(5):395–404. 10.1097/DBP.0000000000000285 27011002 PMC4887346

[52] Wulff K Dijk DJ Middleton B : Sleep and circadian rhythm disruption in schizophrenia. *Br. J. Psychiatry.* 2012;200:308–316. 10.7554/eLife.05105 22194182 PMC3317037

[53] Wang C Holtzman DM : Bidirectional relationship between sleep and Alzheimer's disease: role of amyloid, tau, and other factors. *Neuropsychopharmacology.* 2020;45(1):104–120. 10.1038/s41386-019-0478-5 31408876 PMC6879647

[54] Iranzo A : Sleep and neurological autoimmune diseases. *Neuropsychopharmacology.* 2020;45(1):129–140. 10.1038/s41386-019-0463-z 31302665 PMC6879573

[55] Valentino RJ Volkow ND : Drugs, sleep, and the addicted brain. *Neuropsychopharmacology.* 2020;45(1):3–5. 10.1038/s41386-019-0465-x 31311031 PMC6879727

[56] Yamazaki S : Extra-SCN Circadian Pacemakers. *Journal of Chronobiology, Japanese Society for Chronobiology.* 2019;25(2):99–111. Reference Source

[57] Pendergast JS Yamazaki S : Effects of light, food, and methamphetamine on the circadian activity rhythm in mice. *Physiol. Behav.* 2014;128:92–98. 10.1016/j.physbeh.2014.01.021 24530262

