# Supplementary material for: Genetics and functional significance of the understudied methamphetamine sensitive circadian oscillator (MASCO)
Source: F1000Res. 2022 Oct 24;11:1018. Originally published 2022 Sep 8. [Version 2] doi: 10.12688/f1000research.125432.2 (PMC9539084; doi:10.12688/f1000research.125432.2)
Supplement: Peer review history

# Peer review: Reviewer response for version 2

**DOI:** 10.5256/f1000research.139870.r154004

**Published:** 2022-11-07

**Authors:** Eric Zhang

**Affiliations:** 1 National Institute of Biological Sciences (NIBS), Beijing, China

My concerns have all been addressed.

Is the review written in accessible language?

Yes

Are all factual statements correct and adequately supported by citations?

Yes

Are the conclusions drawn appropriate in the context of the current research literature?

Yes

Is the topic of the review discussed comprehensively in the context of the current literature?

Yes

Reviewer Expertise:

circadian clock, sleep, mood disorders

I confirm that I have read this submission and believe that I have an appropriate level of expertise to confirm that it is of an acceptable scientific standard.

# Peer review: Reviewer response for version 1

**DOI:** 10.5256/f1000research.137735.r149940

**Published:** 2022-10-06

**Authors:** Eric Zhang

**Affiliations:** 1 National Institute of Biological Sciences (NIBS), Beijing, China

This manuscript summarizes the characteristic of MASCO which induced by methamphetamine in detail and clearly, points out the conditions under which MASCO should be investigated, and provides comprehensive overviews of phenomenon of MASCO in rodents, particularly, with kinds of chemical treatment and different genetic canonical circadian genes knockouts. This is a very well-written review containing detailed summaries and discussions which merit an imminent publication, and will surely promote research in related fields. However, several minor points need to be addressed and certain statements require further justification.

 Points in details:

1. Proposed functional significance of MASCO. Considering the phenomenon that the period length is different in DD and LL condition with methamphetamine, but it is well-known that the locomotor activity rhythm to the light-dark cycle is entrained by SCN. Thus, it is necessary to explore the impact from external cues, such as light. By the way, we know that restricted feeding would like to induce dissociation of the MASCO and SCN activity rhythms during short-term methamphetamine treatment in 2014, it is perhaps also worth illustrating the relationship between food-entrainable oscillator and MASCO.
2. Possible roles for MASCO in human disorders. The manuscript only discussed two human disorders which are N24SWD and ADHD, which is not enough, in my opinion, to cover all roles of MASCO in human disorders. My recommendation is to restructure this part to include more evidence and possibility (for example: the roles of methamphetamine in R6/2 and Q175 Huntington’s disease model mice).
3. Discovery of the extra-SCN pacemaker that is sensitive to methamphetamine. “There are also circadian pacemakers that control circadian rhythms of locomotor activity in rodents, but do not require the SCN or the canonical circadian molecular timekeeping mechanism. These extra-SCN circadian pacemakers are revealed by methamphetamine/amphetamine, restricted food availability (the food-entrainable oscillator or FEO), or other rewarding stimuli such as wheel-running activity and palatable meals”. These descriptions and “fig2” still cited author’s own review ” Extra-SCN Circadian Pacemakers” which published in 2017, it is in need of further discussion and modification.
4. Genetic factors that influence the period of MASCO. “There are three possible reasons that methamphetamine has no apparent effect on activity rhythms in C3H mice.” More detailed arguments are needed here for the sensitivity of C3H mice to methamphetamine because MASCO is of short duration.

Is the review written in accessible language?

Yes

Are all factual statements correct and adequately supported by citations?

Yes

Are the conclusions drawn appropriate in the context of the current research literature?

Yes

Is the topic of the review discussed comprehensively in the context of the current literature?

Yes

Reviewer Expertise:

circadian clock, sleep, mood disorders

I confirm that I have read this submission and believe that I have an appropriate level of expertise to confirm that it is of an acceptable scientific standard.

# Response

**Published:** 2022-10-14

**Authors:** Shin Yamazaki

**Affiliations:** UT Southwestern Medical Center, USA

We thank the reviewer for the supportive comments and suggestions.

1. Thank you for these suggestions. We added a sentence discussing the possibility that FEO and MASCO use the same timekeeping mechanism and are the same oscillator.
2. Thank you for this suggestion. We added a discussion of the DUO as a possible model of bipolar disorder. Additionally, we added further discussion of schizophrenia.  
3. We added a sentence that FEO and MASCO may use the same mechanism to generate the rhythm.
4. We added a sentence that the genetic factors causing a short MASCO period in C3H mice is unknown.

# Peer review: Reviewer response for version 1

**DOI:** 10.5256/f1000research.137735.r150555

**Published:** 2022-09-20

**Authors:** Ralph Mistlberger

**Affiliations:** 1 Department of Psychology, Simon Fraser University, Burnaby, BC, Canada

This is a well-organized and clearly written review of a fascinating phenomenon, whereby chronic ingestion of methamphetamine alters and/or induces circadian or quasi-circadian rest-activity rhythms in rodents. The article provides a good historical overview of studies on the phenomenon, and a cogent summary of the properties of these rhythms under different environmental conditions and in mouse lines with various clock gene mutations. I have a few suggestions for additional content:

1. Abstract: “We discuss these fundamental properties of MASCO and synthesize studies of strain, sex, and circadian gene mutations on MASCO”. I suggest deleting the word ‘synthesize’, or replacing it with ‘integrate’ or some other word.
2. Abstract: ‘Canonical’. Can we all stop using that word so much in reference to clock genes? Students often find it confusing, as if it refers to a particular type of gene.
3. Proposed functional significance of MASCO. “One major function of the SCN is to orchestrate the phase of peripheral circadian oscillators.39–41 The Menaker group showed that MASCO can at least partially substitute for this SCN function.” ****MASCO organizes rest-activity states, which determine when animals are awake to feed, which determines the daily rhythm of food intake. It would be worth noting that food intake may be the periodic stimulus that mediates synchrony of peripheral oscillators with rest-activity rhythms induced by methamphetamine intake. MASCO may be at the top of a hierarchy, but may in fact only control behaviour, and have no direct output to peripheral oscillators.
4. Proposed functional significance of MASCO. “The researchers concluded that there are two circadian oscillators in human, one that controls physiology (core body temperature and melatonin) with a period close to 24 h and another that controls the sleep-wake cycle with periods that significantly deviate from 24 h (either extremely longer or shorter than 24 h).” This phenomenon, in humans, has also been modelled as an artefact related to changes in time perception and to instructions not to nap. When naps did occur, they weren’t included in the actograms. When they were added to the actograms (Zulley and Campbell), the evidence for desynchrony disappeared (subjects slept at the temperature minimum most if not every cycle), at least in some of the published records for which nap data were available. Aschoff did not dispute this but did point out that there was a change in time perception, and meal timing etc. suggesting a prolonged biological day. The change in time perception could be independent of circadian timing, and related instead to an effect of temporal isolation.
5. I’m surprised that the authors don’t discuss the possible relationship between MASCO and food-entrainable oscillators thought to drive food anticipatory activity rhythms in animals on daily feeding schedule. Both types of rhythms are SCN dependent, and seem robust to various clock gene knockouts, and may involve dopamine signalling pathways. I don’t believe that there is any compelling evidence ruling out the possibility that the two are manifestations of the same timing mechanism.
6. Possible roles for MASCO in human disorders. I’m also surprised that the DUO model of bipolar disorder, proposed by Blum and Storch (citation 16) isn’t discussed here.

Is the review written in accessible language?

Yes

Are all factual statements correct and adequately supported by citations?

Yes

Are the conclusions drawn appropriate in the context of the current research literature?

Yes

Is the topic of the review discussed comprehensively in the context of the current literature?

Yes

Reviewer Expertise:

Chronobiology and sleep research

I confirm that I have read this submission and believe that I have an appropriate level of expertise to confirm that it is of an acceptable scientific standard.

# Response

**Published:** 2022-10-14

**Authors:** Shin Yamazaki

**Affiliations:** UT Southwestern Medical Center, USA

We thank the reviewer for the supportive and insightful comments.

  

1. We replaced ‘synthesize’ with ‘integrate.’
2. In the abstract, we removed ‘canonical’ and simply said ‘circadian genes.’
3. We added this explanation as a possible mechanism by which MASCO coordinates the phases of peripheral oscillators.
4. Thank you for bringing this to our attention. We were not aware of the possible artefact caused by instructions not to nap and the impact of changes in time perception. We reviewed this study and added the caveats to this paragraph.
5. The reason we didn’t mention this is that we discuss this topic in our prior review articles. However, we agree this is important and therefore mentioned that the extra-SCN oscillators may utilize the same timekeeping mechanism and may be the same oscillator.  
6. Thank you for this suggestion. We have discussed that Blum and Storch proposed the DUO as a model of bipolar disorder.
